# Microarray-based comparative genomic profiling of reference strains and selected Canadian field isolates of *Actinobacillus pleuropneumoniae*

**DOI:** 10.1186/1471-2164-10-88

**Published:** 2009-02-24

**Authors:** Julien Gouré, Wendy A Findlay, Vincent Deslandes, Anne Bouevitch, Simon J Foote, Janet I MacInnes, James W Coulton, John HE Nash, Mario Jacques

**Affiliations:** 1 Groupe de Recherche sur les Maladies Infectieuses du Porc, Université de Montréal, St-Hyacinthe, Québec J2S 7C6, Canada; 2 Institute for Biological Sciences, National Research Council of Canada, Ottawa, Ontario K1A 0R6, Canada; 3 Department of Pathobiology, Ontario Veterinary College, University of Guelph, Guelph, Ontario N1G 2W1, Canada; 4 Department of Microbiology and Immunology, McGill University, Montréal, Québec H3A 2B4, Canada; 5 Centre de Recherche en Infectiologie Porcine, Université de Montréal, St-Hyacinthe, Québec J2S 7C6, Canada; 6 Office of Biotechnology, Genomics and Population Health, Public Health Agency of Canada, Ottawa, Ontario K1A 0K9, Canada

## Abstract

**Background:**

*Actinobacillus pleuropneumoniae*, the causative agent of porcine pleuropneumonia, is a highly contagious respiratory pathogen that causes severe losses to the swine industry worldwide. Current commercially-available vaccines are of limited value because they do not induce cross-serovar immunity and do not prevent development of the carrier state. Microarray-based comparative genomic hybridizations (M-CGH) were used to estimate whole genomic diversity of representative *Actinobacillus pleuropneumoniae *strains. Our goal was to identify conserved genes, especially those predicted to encode outer membrane proteins and lipoproteins because of their potential for the development of more effective vaccines.

**Results:**

Using hierarchical clustering, our M-CGH results showed that the majority of the genes in the genome of the serovar 5 *A. pleuropneumoniae *L20 strain were conserved in the reference strains of all 15 serovars and in representative field isolates. Fifty-eight conserved genes predicted to encode for outer membrane proteins or lipoproteins were identified. As well, there were several clusters of diverged or absent genes including those associated with capsule biosynthesis, toxin production as well as genes typically associated with mobile elements.

**Conclusion:**

Although *A. pleuropneumoniae *strains are essentially clonal, M-CGH analysis of the reference strains of the fifteen serovars and representative field isolates revealed several classes of genes that were divergent or absent. Not surprisingly, these included genes associated with capsule biosynthesis as the capsule is associated with sero-specificity. Several of the conserved genes were identified as candidates for vaccine development, and we conclude that M-CGH is a valuable tool for reverse vaccinology.

## Background

*Actinobacillus pleuropneumoniae *is a Gram-negative bacterium belonging to the family *Pasteurellaceae*. It is the etiological agent of porcine pleuropneumonia, a highly contagious respiratory infection that causes severe economic losses to the swine industry worldwide. The disease, transmitted by the aerosol route or direct contact with an infected pig, is characterized by hemorrhagic, fibrinous and necrotic lung lesions [1-3]. The clinical picture may range from peracute to chronic and asymptomatic carrier pigs can transmit the disease when introduced into uninfected herds. Two different biovars are recognised within the species: biovar 1 strains are nicotinamide adenine dinucleotide (NAD) dependent while biovar 2 strains are NAD-independent [4,5]. Based on capsular polysaccharides and lipopolysaccharide (LPS) O-chain components, 15 serovars have been described. Serovars 1 to 12 and 15 usually belong to biovar 1; whereas serovars 13 and 14 isolates are typically biovar 2 [5-8], however, biovar 2 variants of serovar 2, 4, 7 and 9 have been reported [5,9,10].

Serotyping and other genetic typing methods for *A. pleuropneumoniae *have contributed greatly to surveillance and epidemiological studies. These tools provide important information for decision making in control programs aimed at eradication of virulent types of the bacterium. Nevertheless, serological cross-reactivity between *A. pleuropneumoniae *serovars 1, 9 and 11 [11], between serovars 3, 6 and 8 [12], and between 4 and 7 [13] has been described. In North America, serovars 1, 5 and 7 are reported to be the most prevalent, while serovars 2 and 9 are most commonly isolated in Europe, and serovar 15 is the predominant isolate from Australian pigs [14-16].

The virulence factors described for *A. pleuropneumoniae *include LPS, capsular polysaccharides, Apx toxins (I-IV), outer membrane proteins (OMPs) and various iron acquisition systems. However, the overall contribution of each component to the infection process remains unclear, as do the mechanisms of pathogenesis of this organism [16-18]. All serovars are capable of causing disease; however, some serovars such as serovars 1, 2, 5a, 5b, 9 and 11 are more frequently involved in severe outbreaks with high mortality and pulmonary lesions. Several reports have suggested that the differences in virulence among the serovars can mainly be attributed to different combinations of Apx toxins and the amount of capsular polysaccharides [17,19-21]. Almost all of the currently available vaccines against *A. pleuropneumoniae *are either inactivated whole-cell bacterins or subunit combinations of Apx toxins and proteins or OMPs [22]. Experimental challenge and field usage data indicate that these vaccines neither induce cross-serovar immunity nor prevent development of the carrier state and have little impact on morbidity [23-26].

Molecular techniques, including Multilocus Enzyme Electrophoresis (MLEE) [27,28], Pulsed-Field Gel Electrophoresis (PFGE) [29] and Amplified Fragment Length Polymorphism (AFLP) analysis [30] have been used to study different strains of *A. pleuropneumoniae*. These investigations have shown that genetic diversity among isolates of the same serovar may be almost equivalent to that in the species as a whole, suggesting that *A. pleuropneumoniae *strains are genetically very similar and would seem to have a clonal population structure. However, restriction analysis fingerprinting [31,32] studies revealed that with the exception of serovars 1 and 9, the reference strains of *A. pleuropneumoniae *are clearly different. Similarly, work of Chevallier *et al. *and Møller *et al. *revealed a more pronounced heterogeneity in the chromosomal structure among strains of serovars 1, 5a, 5b, 7, 8 and 12 [27,29]. The notion of heterogeneity amongst serovars is also supported by early free-solution DNA-DNA hybridization studies that showed that representative strains of 12 serovars of *A. pleuropneumoniae *shared 74 to 90% sequence homology with *A. pleuropneumoniae *serovar 1 [32].

We are using a reverse vaccinology approach to identify new candidates for the development of cross-protective vaccines against *A. pleuropneumoniae*. Using the complete and annotated *A. pleuropneumoniae *L20 genome [33], Chung and coworkers published a list of 93 predicted OMPs or lipoproteins of *A. pleuropneumoniae *obtained by using five genome scanning programs [34]. The availability of the genome sequence of *A. pleuropneumoniae *also enables us to study diversity of *A. pleuropneumoniae *on a genome-wide scale. To date, two additional complete genomic sequences have become available, *A. pleuropneumoniae *serovar 3 JL03 [GenBank:CP000687] [35], and *A. pleuropneumoniae *serovar 7 AP76 [GenBank:CP001091].

M-CGH is a powerful tool to estimate whole genomic diversity and to study the gene content and locate genomic islands in closely related strains of bacteria [36-42]. In the present study, this method was applied for the first time to study genetic relationships among reference strains of the 15 serovars of *A. pleuropneumoniae *and representative field isolates. Our goal was to identify conserved genes with particular emphasis on those predicted to encode outer membrane proteins and lipoproteins because of their potential for the development of improved vaccines.

## Results and Discussion

**Figure 1:**
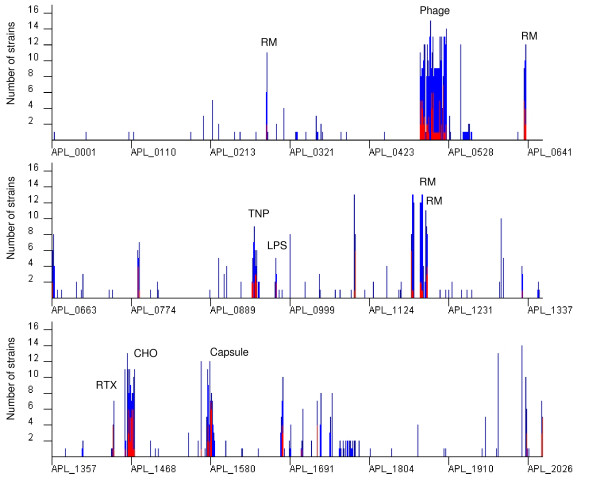
**Number of reference strains representing the 15 serovars of *A. pleuropneumoniae *where gene is divergent or missing for each gene, ordered as in *A. pleuropneumoniae *L20 genome sequence and based on M-CGH results**. (Blue: -3<Log2Ratio<-1, Red: Log2Ratio<-3). RM, DNA restriction/modification enzymes; TNP, transposon; LPS, lipopolysaccharide biosynthesis genes; RTX, toxin genes; CHO, carbohydrate biosynthesis genes.

The first microarray-based study of *A. pleupneumoniae*, which used a full-genome microarray based upon a draft version of the genome sequence of strain L20, evaluated the effects of iron limitation [43]. From the recently completed genome sequence of *A. pleuropneumoniae *L20 [33], a full genome *A. pleuropneumoniae *microarray (AppChip2; GEO Accession Number GPL6658), which takes into account corrections from the draft sequence and comprises reporters matching the sequence of more than 1800 genes, was developed and used in this study. With microarray hybridization, the presence of a specific gene in a test strain is based on comparison of the intensity of the hybridization signal obtained with the genomic DNA of the tester strain to that obtained with the genomic DNA of the control strain for the corresponding reporter. The ratio of intensity of tester signal to control signal is usually expressed on a log2 scale and we used a threshold of -1 to define genes likely to be divergent in sequence from the strain L20 genome. We have shown previously that genes absent from the tested strain usually have log2 ratio values less than -3 [37]. The overall genomic variability of the 15 *A. pleuropneumoniae *serovars is shown in Figure 1 where the number of serovars in which a gene is variable is plotted for each gene with the gene order corresponding to the strain L20 genome sequence. Although most genes are conserved across the 15 serovars, we observed a number of distinct clusters of absent/divergent genes. A total of 205 genes were identified as either divergent or highly divergent/absent in the 15 reference strains tested (additional file 1). In the largest cluster (APL_0488 to APL_0525), many genes are annotated as potential phage or prophage genes, suggesting that this cluster might correspond to a phage. A smaller cluster around APL_0947 to APL_0952 corresponds to genes annotated as transposon-related. Several of the other clusters correspond to genes annotated as components of DNA restriction and modification systems. Clusters of variable genes involved in toxin production, and in capsule and LPS biosythesis were observed as expected. Several other clusters containing unannotated genes may warrant further investigation. Experiments with closely related bacterium *Actinobacillus suis *showed only weak hybridization to the AppChip2 microarray, suggesting that the nucleotide sequence identities for the ORFs are low; consistent with earlier DNA-DNA hybridization studies [32] (data not shown).

**Figure 2:**
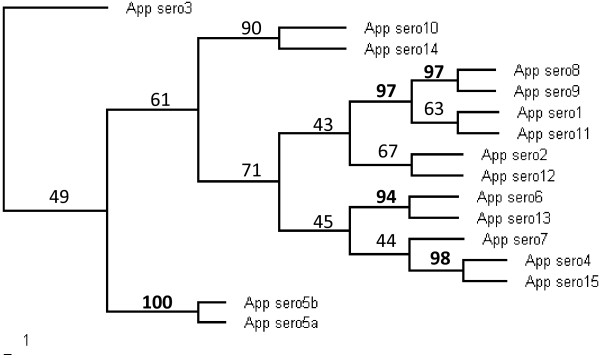
**Hierarchical clustering of *A. pleuropneumoniae *reference strains based on M-CGH data excluding phage and transposase genes**. The dendrogram was produced using the MEV software from the J. Craig Venter Institute with Euclidean distance and average linkage clustering (n = 1000 bootstrap iterations).

We used hierarchical clustering based on the M-CGH results to examine the relationship between *A. pleuropneumoniae *serovars based on genomic content. The dendrogram of the data excluding phage and transposon-related genes is shown in Figure 2. This tree has very similar structure to the tree based on data from all 1857 genes on AppChip2 (data not shown), and both show serovars 5a and 5b forming a distinct subclade. Also, the antigenically related serovars 1, 11, and 9 cluster together as do cross-reactive serovars 4 and 7. Nevertheless we do not observe clustering of serovars 1, 5 and 7 which are the most common ones found in North America or of serovars 13 and 14 which represent biovar 2.

**Figure 3:**
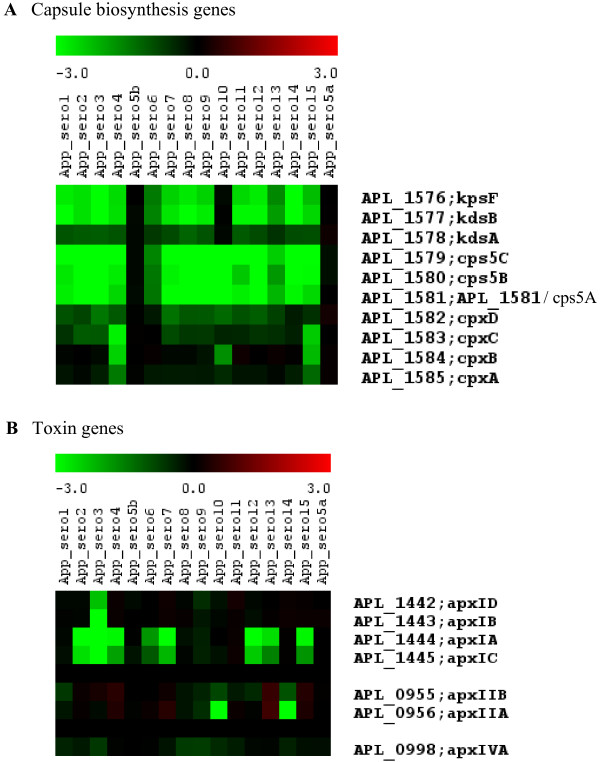
**Variability of genes involved in toxin and capsule biosynthesis across the *A. pleuropneumoniae *reference strains representing the 15 serovars**. Green indicates that the gene is divergent in sequence or absent in the tester strain.

The M-CGH patterns for capsule biosynthesis genes (Figure 3A) show a high degree of variability for the various serovars compared to serovar 5b. This is to be expected as the capsule is a major determinant of the *A. pleuropneumoniae *serovar [6,44]. Much less variation across serovars is observed with the *cpx *genes which are involved in capsule export than with the *cps *and *kds *genes which are involved in capsule biosynthesis.

Serotyping of *A. pleuropneumoniae *is based mainly on the capsular polysaccharide (CPS) and the lipopolysaccharide antigenic O-chain component [44]. The M-CGH patterns for capsule biosynthesis genes (Figure 3A) show a high degree of variability for the various serovars compared to serovar 5b, except for serovar 5a. The structures of CPS from subtypes 5a and 5b have been determined [45,46]. Both structures share a common backbone consisting of disaccharide repeating units, [→6)-α-D-Glc_p_NAc(1→5)-β-KDO_p_-(2→]_n_. In addition, the CPS of serovar 5b has a lateral β-D-glucopyranosyl residue [45]. Moreover, both subtypes contain LPS 0-chain components with the same basic polysaccharide structure of a linear unbranched homopolymer of 1,6-linked β-D-galactopyranosyl residues [47]. Thus, the related structures of the capsular polysaccharides of subtypes 5a and 5b are consistant with the identical M-CGH patterns for capsule biosynthesis genes for these two serovars.

The M-CGH pattern of toxin biosynthesis genes for the reference strains of the 15 serovars is shown in Figure 3B. The pattern observed for the toxin genes is in agreement with the results of Frey and co-workers [48] who reported that the *apxIDB *genes are missing in serovar 3; *apxIAC *are missing in serovars 2, 3, 4, 6, 7, 12, 13 and 15; *apxIIAB *genes are missing in serovars 10 and 14. Consistent with previous reports [49-51], *apxIVA *was present in all serovars; as *apxIII *genes are not present in *A. pleuropneumoniae *serovar 5 strains (including L20) they were therefore not included on the AppChip2 microarray.

**Figure 4:**
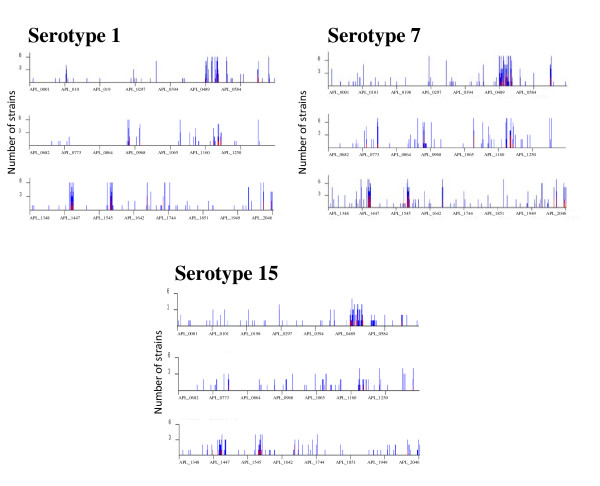
**Number of strains from 15 fresh field isolates of *A. pleuropneumoniae *serovars 1, 7 and 15 where the sequence is divergent or absent for each gene, ordered as in *A. pleuropneumoniae *L20 genome sequence and based on M-CGH results**.

The genomic variability of 15 representative field isolates of *A. pleuropneumoniae *serovars 1, 7 and 15 is shown in Figure 4. For each serovar, the number of strains in which a gene is variable is plotted for each gene with the gene order corresponding to the strain L20 genome sequence. For serovars 5a, we observed only one cluster of highly variable genes which is the largest cluster (APL_0488 to APL_0525) containing many genes annotated as potential phage or prophage genes in serovar 5b.

**Figure 5:**
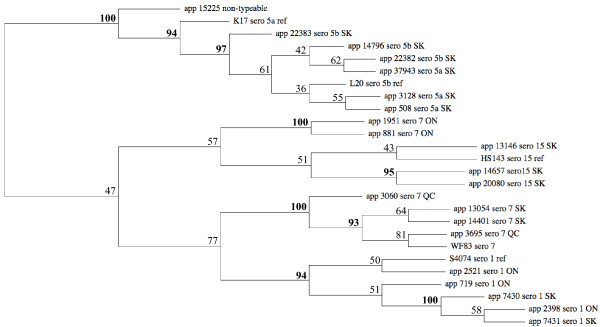
**Hierarchical clustering of field isolates of *A. pleuropneumoniae *based on M-CGH data excluding phage and transposase genes**.

To evaluate relationships among the 21 field isolates of serovars 1, 5, 7 and 15 and their respective reference strains, we performed hierarchical clustering to build dendrograms based upon analysis of the data excluding phage and transposase genes (Figure 5). Except for serovar 7, strains from the same serovar formed a cluster. The analysis indicated that the Ontario serovar 7 field isolates 881 and 1951 clustered separately from the Quebec and Saskatchewan serovar 7 strains. In contrast, Ontario and Saskatchewan serovar 1 field isolates were genetically very similar. As expected, the eight serovar 5a and 5b strains form a distinct cluster. These results are consistent with earlier restriction endonuclease fingerprinting analysis, which revealed limited heterogeneity amongst isolates of serovar 1 or serovar 5 whereas serovar 7 isolates showed greater variation [31].

In Canada, the most prevalent serovars are 1, 5 and 7. However, other serovars have also been isolated from sporadic outbreaks of pleuropneumonia. This is the first report describing the isolation and characterization of serovar 15 strains from field cases of porcine pleuropneumonia in North America. In previous reports, serovar 15 strains have only been isolated from pigs in Australia and Japan [15,52]. Hierarchical clustering based on our M-CGH results showed that the three serovar 15 field strains (05–13146, 05–14657, 05–20080) are closely related to the reference strain of serovar 15 of *A. pleuropneumoniae*. These three serovar 15 field strains also had the same M-CGH pattern of toxin biosynthesis genes as reported for serovar 15 field strain isolated in Japan [52] and the reference strain (HS143). In these strains the Apx structural genes, *apxIIA*, *apxIIB *and *apxIVA*, but not *apxIA *and *apxIC*, and Apx secretion genes *apxIBD *were detected (data not shown).

The serologically non-typeable isolate, 05–15225, possessed the same M-CGH pattern of toxin biosynthesis genes as the reference strains of serovars 5a and 5b. The M-CGH pattern for capsule biosynthesis genes of the 05–15225 isolate showed little variability compared to serovar 5b. Only two genes involved in capsule biosynthesis, *cpsC *and *cpsD *had a log2 ratio value less than -1. These two genes may be deleted or diverged in sequence in this isolate, which could explain why it was not typable by serology. Nevertheless, the dendrogram showed that 05–15225 isolate is closely related to the serovar 5 (subtypes a and b), suggesting that it can be classified in serovar 5 strains.

Other clustering methods or distances were applied to the data set in order to verify that the clusters we identified were robust (data not shown). Hierarchical clustering with Manhattan Distance metrics and K-Medians Clustering (K-MC) [53] yielded highly similar results: strains from the same serovar clustered together, with the serovar 7 strains from Ontario grouped with serovar 15 strains, although the serotype 15 13146 strain was left on its own following K-Medians Clustering. In both cases, the non-typeable 05–15225 isolate clustered with serovar 5 strains. Cluster Affinity Search Technique [54] generated the same results, however serovar 15 strains were scattered in three different clusters, and were not grouped with serovar 7 strains from Ontario.

Commercially available vaccines against *A. pleuropneumoniae*, including inactivated whole-cell bacterins and subunit vaccines, have limited efficacy and little impact on morbidity. Moreover, these vaccines confer only partial protection against the homologous serovar and generally do not confer protection against challenge with heterologous serovars [55-57]. A major focus of research for the development of new vaccines against porcine pleuropneumonia has been to identify proteins that are conserved in all 15 serovars of *A. pleuropneumoniae *and that generate cross-protection against strains of all serovars. Based on the principle that surface-exposed antigens are more susceptible to recognition by antibodies and therefore are the most suitable candidates for a vaccine, the full genome of *A. pleuropneumoniae *L20 was screened using bioinformatics predictor programs to identify open reading frames encoding putative proteins localized at the bacterial outer membrane and 45 OMPs and 48 lipoproteins were predicted [34]. Using M-CGH to identify genes that are highly conserved among the reference strains of the 15 serovars of *A. pleuropneumoniae*, as well as among field isolates, we identified 58 potential vaccine targets (24 OMPs and 34 lipoproteins) (Table 1) that are conserved among all serovars and biovars. Among these candidates, four have been shown to be expressed *in vivo*. Using selective capture of transcribed sequences analysis (SCOTS) [58], it has been shown that *ompA *(*APL_1421*) and *APL_0829 *are expressed by *A. pleuropneumoniae *in necrotic pig lung tissue. Furthermore, polyamine transport protein D (APL_0368), Omp P2 (APL_0649), OmpA (APL_1421) and outer membrane antigenic lipoprotein B (APL_1930) are required for efficient colonization of the porcine host by *A. pleuropneumoniae *as shown by signature-tagged mutagenesis (STM) experiments [59,60].

**Table 1:** *A. pleuropneumoniae *reference strains and field isolates analyzed by M-CGH in this study.

Strains	Serovars	Source
*Reference strains*		All from K.R. Mittal^1^
S4074	1	
S1536	2	
S1421	3	
M62	4	
K17	5a	
L20	5b	
Femø	6	
WF83	7	
405	8	
CVJ 13261	9	
13069	10	
56153	11	
8329	12	
N273	13	
3906	14	
HS143	15	

*Field strains*		
05–7430, 05–7431	1	M. Ngeleka ^2^
719, 2398, 2521	1	D. Slavic^3^
04–37943, 04–3128, 05–508	5a	M. Ngeleka ^2^
04–14796, 03–22382, 03–22383	5b	M. Ngeleka ^2^
05–3695, 06–3060	7	S. Messier^1^
04–13054, 05–14401	7	M. Ngeleka ^2^
881, 1951	7	D. Slavic^3^
05–13146, 05–14657, 05–20080	15	M. Ngeleka ^2^
05–15225	non-typeable	M. Ngeleka ^2^

^1 ^Faculté de médecine vétérinaire, Université de Montréal, St-Hyacinthe, QC

^2 ^Prairie Diagnostic Services, University of Saskatchewan, Saskatoon, SK

^3 ^Ontario Veterinary College, University of Guelph, Guelph, ON

*A. pleuropneumoniae *bind preferentially to cells of the lower respiratory tract, where some essential nutrients for the growth of bacteria, such as iron, are limited. *A. pleuropneumoniae *has developed several iron uptake systems including the hydroxamate siderophore receptor FhuA (APL_2016), a hemoglobin-binding receptor HgbA (APL_1047) and a transferrin receptor complex composed of two outer membrane proteins, transferrin-binding protein A (TbpA; APL_1567) and transferring-binding protein B (TbpB; APL_1568). Not only is iron essential for growth of bacteria but iron-restriction is an important signal that controls expression of many genes including some coding for virulence factors [43]. Hence, these proteins involved in iron uptake are considered as candidates for development of subunit vaccines. However, our M-CGH results showed that only one protein involved in iron uptake, TbpA, is conserved among the 15 serovars of *A. pleuropneumoniae*. This observation could explain the partial protection against infection with heterologous strains conferred by an acellular pentavalent subunit vaccine containing the TbpB of *A. pleuropneumoniae *serovar 7 [26].

The NADPH-sulfite reductase hemoprotein CysI (APL_1842) of *A. pleuropneumoniae*, a cytoplasmic protein involved in cellular metabolism, has also been shown to induce protective immunity against a homologous challenge [61]. Our CGH analysis showed that CysI is conserved among all strains tested in this study, thus this protein could represent an interesting vaccine target.

Two genes (*APL_1421 *and *APL_1852*), encoding homologs of outer membrane protein A (or OMP P5), were identified as potential vaccine targets in this study. APL_1421 and APL_1852 have 70.6% identity and showed 75.6% and 70.6% identity, respectively, to OmpA from the bovine pathogen *Mannheimia haemolytica*, which has surface exposed epitopes and is recognized by convalescent bovine sera [62,63]. In addition to *ompA *(*APL_1421*), the outer-membrane lipoprotein LolB (APL_0777), is expressed *in vivo *by *A. pleuropneumoniae *and they have been identified and characterized as potential components of a cross-protective sub-unit vaccine against *A. pleuropneumoniae *[64]. However, Oldfield *et al. *reported that neither of these proteins was capable of eliciting protective immunity against *A. pleuropneumoniae *challenge [64]. The outer membrane protein PalA (APL_0304) was also on our list of conserved proteins. Nevertheless, it has been shown that vaccination with PalA increases the severity of *A. pleuropneumoniae *infection in vaccinated pigs [65]. Thus, the deleterious effect of PalA in vaccination and the inability of LolB and OmpA to induce protective immunity eliminate these proteins from the list of potential vaccine candidates.

Interestingly, homologs of some of the remaining conserved vaccine candidates are already under investigation as vaccine components in other bacteria (including other Pasteurellaceae). For example, the lipoprotein Plp4 from *M. haemolytica*, which had 85.3% identity with lipoprotein PlpD (APL_0460), was identified by screening antigens of *M. haemolytica *with sera from Presponse™ vaccinated calves (a cell-free culture supernatant *M. haemolytica *A1 vaccine) that were protected from *M. haemolytica *A1 infections [66], thus suggesting PlpD may be a protective antigen. Another candidate, APL_0378, a glycerophosphodiester phosphodiesterase (GlpQ), showed 82% and 79.5% identity to GlpQ of *Pasteurella multocida *and protein D of *Haemophilus influenzae*, respectively. The role of GlpQ in *A. pleuropneumoniae *and *P. multocida *is unknown, however, its homolog in *H. influenzae *has been shown to mediate the acquisition of choline directly from the membranes of epithelial cells in culture and incorporate it into its own LPS [67]. *H. influenzae *protein D has been showed to elicit cross-protection against virulent heterologous strains of *H. influenzae *in rats [68]. In contrast to *H. influenzae*, GlpQ in *P. multocida *is not surface-exposed and is unable to stimulate protective immunity, even though vaccinated animals have high antibody titers [69]. Therefore, the location of GlpQ and accessibility of GlpQ-specific antibodies should be determined in *A. pleuropneumoniae*.

OMP P4 (*ompP4*; ALP_0389) is another attractive surface exposed antigen [70] showing 65% identity to lipoprotein E (also known as OMP P4) in *H. influenzae*, which is highly conserved among both typeable and nontypeable strains [71]. In nontypeable strains of *H. influenzae *(NTHi), OMP P4 is essential for utilization of NAD and subsequent growth [72,73]. Intranasal immunization of mice with OMP P4 in a mucosal adjuvant induces protective immune responses against NTHi infections and notably, a mucosal immune response, which reduces NTHi nasopharynx colonization [74]. These observations in *H. influenzae *suggest that lipoprotein E (APL_0389) may be an attractive candidate for a vaccine against *A. pleuropneumoniae*.

PCP (peptidoglycan-associated lipoprotein cross-reacting protein) of *H. influenzae *is under investigation since it is surface-exposed and anti-PCP serum shows bactericidal activity against several clinical isolates of type b and non-typeable *H. influenzae *[75]. Outer membrane lipoprotein SlyB (APL_0037) shows highest identity (71.4%) to this protein. However, although PCP from *P. multocida *(also surface-exposed) is recognized by convalescent chicken antiserum, it is unable to stimulate protective immunity [69].

The polyamine transport protein D, PotD (APL_0368), has extensive homology to a 38 kDa lipoprotein, Lpp38 of *M. haemolytica*. Lpp38 is surface-exposed and is recognized by sera from calves resistant to infection after natural exposure to *M. haemolytica *and by sera from calves vaccinated with M. *haemolytica *A1 outer membranes or with live bacteria [76]. Recently, in the human pathogen *Streptococcus pneumoniae*, PotD has been reported to be involved in virulence in both an animal model of sepsis and pneumonia [77,78]. Active immunization of mice with recombinant PotD induces a vigorous antibody response and provides a significant degree of protection against lethal pneumococcal infection [79]. These data suggest that PotD plays a role in the development of immunity to bacterial infections and may be a protective antigen.

Homologs of the outer membrane protein D15 (APL_0411) have been reported in several pathogenic bacteria including *H. influenzae*, *Haemophilus ducreyi*, *P. multocida*, *Neisseria meningitidis *and *Shigella dysenteriae *[80-83]. These studies suggest a role for these proteins in pathogenesis and immunity. Notably, it has been shown that D15 confers protection against homologous and heterologous strains of *H. influenzae *in animal models [84-86]. Similarly, Oma87, a closely related homolog in *P. multocida*, has been shown to elicit protection in animal model of infection [83]. Thus D15 is potentially an attractive vaccine target.

## Conclusion

In this study, we have shown that M-CGH can be a useful tool to identify candidates for reverse vaccinology in order to develop subunit vaccines to *A. pleuropneumoniae*. After comparing CGH data for 15 reference strains and 21 fresh field isolates, we have identified 58 conserved genes that are predicted to code for outer membrane proteins or lipoproteins. This could assist in development of vaccines with efficacy across serovar boundaries. Future investigations will include the use of microarray transcript profiling experiments of *A. pleuropneumoniae *isolated from infected pigs to identify potential vaccine candidates that are both conserved and expressed *in vivo *during infection in pigs.

## Methods

### Bacterial strains

Reference strains and field isolates of *A. pleuropneumoniae *used in this study are listed in Table 2. All *A. pleuropneumoniae *strains were inoculated into Brain-Heart Infusion (BHI, Difco Laboratories, Detroit, MI) medium supplemented with NAD: either 15 μg/ml in agar or 5 μg/ml in broth. Cultures were grown at 37°C for 16–18 hours before genomic DNA isolation.

**Table 2:** *In silico *predicted OM proteins and lipoproteins from *A. pleuropneumoniae *(according to Chung et al. 2007), which are conserved amongst the 15 reference strains and the 21 field isolates tested by M-CGH in this study.

**OrfID**	**Gene**	**Product**
*Predicted outer membrane proteins*		
APL_0006	*ompP2A*	outer membrane protein P2
APL_0049	*APL_0049*	hypothetical protein
APL_0200	*hofQ*	type II secretory pathway, component HofQ
APL_0245	*APL_0245*	transferrin binding protein-like solute binding protein
APL_0257	*APL_0257*	probable outer membrane protein
APL_0276	*frpB*	iron-regulated outer membrane protein B
APL_0304	*palA*	outer membrane protein precursor PalA
APL_0410	*APL_0410*	hypothetical outer membrane protein
APL_0411	*D15*	protective surface antigen D15 precursor
APL_0460	*plpD*	lipoprotein Plp4
APL_0565	*cirA*	hypothetical ABC transporter ATP-binding protein
APL_0649	*ompP2*	Outer membrane protein P2 precursor (OMP P2)
APL_0829	*APL_0829*	hypothetical protein
APL_0840	*APL_0840*	predicted outer membrane protein
APL_0919	*irp*	iron-regulated outer membrane protein
APL_0959	*APL_0959*	hemagglutinin/hemolysin-like protein
APL_0962	*ostA*	organic solvent tolerance protein precursor
APL_1421	*ompA*	outer membrane protein P5 precursor
APL_1567	*tbpA*	transferrin-binding protein 1 Tbp1
APL_1705	*APL_1705*	FKBP-type peptidyl-prolyl cis-trans isomerase
APL_1815	*APL_1815*	hypothetical protein
APL_1852	*ompA*	Outer membrane protein P5 precursor (OMP P5)
APL_1921	*pgaA*	biofilm PGA synthesis protein PgaA precursor
APL_2002	*APL_2002*	hypothetical protein

*Predicted lipoproteins*		
APL_0029	*APL_0029*	ABC transporter periplasmic protein
APL_0036	*APL_0036*	hypothetical protein
APL_0037	*slyB*	outer membrane lipoprotein
APL_0116	*APL_0116*	hypothetical protein
APL_0124	*APL_0124*	hypothetical protein
APL_0156	*apbE*	thiamine biosynthesis lipoprotein ApbE precursor
APL_0227	*APL_0227*	hypothetical protein
APL_0236	*APL_0236*	putative lipoprotein
APL_0332	*hlpB*	lipoprotein HlpB
APL_0356	*APL_0356*	hypothetical protein
APL_0368	*potD2*	spermidine/putrescine-binding periplasmic protein 1 precursor
APL_0378	*glpQ*	glycerophosphoryl diester phosphodiesterase
APL_0389	*ompP4*	lipoprotein E precursor
APL_0428	*smpA*	small protein A
APL_0603	*APL_0603*	hypothetical protein
APL_0611	*APL_0611*	putative lipoprotein
APL_0642	*mltB*	membrane-bound lytic murein transglycosylase B
APL_0777	*lolB*	outer-membrane lipoprotein LolB precursor
APL_0816	*mltA*	membrane-bound lytic murein transglycosylase A precursor
APL_0873	*rlpB*	putative rare lipoprotein B
APL_0920	*APL_0920*	hypothetical protein
APL_1062	*APL_1062*	hypothetical protein
APL_1121	*APL_1121*	putative lipoprotein
APL_1152	*APL_1152*	hypothetical protein
APL_1273	*APL_1273*	putative fimbrial biogenesis and twitching motility protein PilF-like protein
APL_1297	*APL_1297*	hypothetical protein
APL_1362	*APL_1362*	hypothetical protein
APL_1404	*oapB*	opacity associated protein B
APL_1741	*mltC*	Membrane-bound lytic murein transglycosylase C precursor
APL_1875	*APL_1875*	hypothetical protein
APL_1898	*APL_1898*	hypothetical protein
APL_1913	*pepO*	neutral endopeptidase
APL_1930	*APL_1930*	Outer membrane antigenic lipoprotein B precursor
APL_1957	*APL_1957*	Lipoprotein_5 domain containing protein

### Construction of an A. pleuropneumoniae amplicon-based DNA microarray (AppChip 2)

PCR primers were designed for 1954 genes of the *A. pleuropneumoniae *L20 (serovar 5b) genome using the Primer3 program [87] controlled by an automated script as described previously [make_primers code available at ] [88]. Most sequences of length greater than 2000 nt were split to create two or more reporters corresponding to a single large ORF. Primer selection parameters were standardized and included similar predicted melting temperature (60 ± 2°C), uniform length (25 nt), and minimum amplicon size of 160 bp. Generation of PCR amplicons and fabrication of DNA microarrays were described previously [88]. Details on the construction of this microarray (AppChip2) are available at NCBI (GEO Accession Number GPL6658). The AppChip2 microarray comprises validated amplicons covering > 92% of the ORFs longer than 160 bp in the final *A. pleuropneumoniae *L20 genome sequence (GenBank accession number CP000569).

### Isolation of genomic DNA

*A. pleuropneumoniae *strains were harvested after growth on agar plates for 16–18 h, resuspended in H_2_O, and treated with lysozyme (Roche, Laval, QC) and RNase A (Qiagen, Mississauga, ON) for 10 min at room temperature. The cell suspensions were then digested with proteinase K (MBI Fermentas, Burlington, ON) for 1 h at 37°C, and complete lysis was obtained by addition of sodium dodecyl sulfate to a final concentration of 0.1% (wt/vol). Genomic DNA, extracted from the cell lysates by two extractions with phenol-chloroform-isoamyl alcohol (25:24:1) and two extractions with chloroform, was precipitated in ethanol.

### Genomic DNA labelling

Isolated genomic DNA was fragmented by nebulization. One hundred μg of DNA in H_2_O and 35% glycerol (v/v) was placed in an AeroMist Nebulizer chamber (IPI Medical Products, Chicago, IL), and sheared by passing nitrogen gas through the chamber at 15 psi for 1 min. The DNA was precipitated with ethanol and suspended in 100 μl of ddH2O. Typically, the DNA was fragmented to a range of 0.4 to 12 kb in size. Five μg of fragmented DNA were fluorescently labeled using direct chemical coupling with the Label-IT (Mirus Corp., Madison, WI) cyanine dyes Cy3 and Cy5 as recommended by the manufacturer. Probes were purified from unincorporated dyes by passing samples through Qiaquick columns (Qiagen, Mississauga, ON). Labeled DNA sample yields and dye incorporation efficiencies were assessed using a Nanodrop ND-1000 spectrophotometer (Nanodrop, Rockland, DE).

### Microarray hybridizations

The hybridization profile for each strain was obtained by co-hybridizing labeled DNA from the tester strain with labeled DNA from the *A. pleuropneumoniae *serovar 5b (L20) control strain to the microarray. DNA from tester strains was labeled with Cy3 and DNA from the control strain with Cy5. Dye swaps were performed on selected strains to test for any dye-incorporation bias. Labeled samples were normalized by selecting tester/control sample pairs with similar dye incorporation efficiencies. Equivalent amounts (2 μg) of labeled tester and control samples were pooled, lyophilized, and then re-suspended in 42 μl of hybridization buffer [1 × DIGEasy hybridization solution (Roche Applied Science); 0.5 μg/μl of *Torulla *yeast tRNA (Invitrogen); 0.5 μg/μl of salmon sperm genomic DNA (Invitrogen)]. Labeled gDNA was denatured at 65°C for 5 min and applied to the microarray. Hybridizations were performed overnight at 37°C under 22 × 40-mm glass cover slips in a high-humidity chamber. Microarrays were washed 2 × 5 min at 50°C in 1 × SSC with 0.1% SDS, then 2 × 5 min at 50°C in 0.5 × SSC, and 1 × 5 min at 50°C in 0.1 × SSC. Slides were spun dry (500 × g, 5 min) and stored in lightproof containers until scanned.

### Data acquisition and analysis

After hybridization with labeled gDNA, microarray slides of the 15 reference serovars were scanned using a Chipreader laser scanner (BioRad, Mississauga, ON) according to the manufacturer's recommendations. Spot quantification, signal normalization and data visualization were performed using ArrayPro Analyzer v4.5 (Media Cybernetics, Silver Spring, MD). Net signal intensities were obtained by performing local-ring background subtraction. "Tester signal" is defined as the signal intensity of the selected *A. pleuropneumoniae *reference strains labeled with appropriate fluorescent dye, while "control signal" is defined as the signal intensity of *A. pleuropneumoniae *strain 5b labeled with its appropriate fluorescent dye. The ratio of tester signal to control signal for each gene was transformed to its base 2 logarithm [89], log_2 _[Tester Signal/Control Signal], and is referred to as "Log2Ratio". Data from each channel were adjusted using cross-channel Loess normalization of the Log2Ratio data and low intensity and anomalous spots were flagged and removed. Data were stored and archived using the BASE BioArray Software Environment [90]. Microarray data from sets of hybridizations were exported from BASE after removal of flagged spots, Loess normalization, and averaging of data from duplicate spots on the microarray. At least three replicates of each strain were performed and the results averaged.

The microarrays for the *A. pleuropneumoniae *field strains were scanned with a Perkin-Elmer ScanArray Express scanner according to the manufacturer's recommendations. Image and data analysis were performed using TM4 suite of softwares from the J. Craig Venture Institute [91]. Raw data were generated using Spotfinder v.3.1.1. The integrated intensities of each spot, equivalent to the sum of unsaturated pixels in a spot were quantified and the integrated intensity of the local background was subtracted for each spot. The same operation was performed with the median spot intensities. Data were normalized with the MIDAS software tool using cross-channel Loess normalization. Spots with median intensities lower than 1000 were removed from the normalized data set. Intensities for duplicate spot were merged to generate the final normalized data set. The results were analyzed using the MEV software, first to check similarity of patterns of gene divergence within replicates for each serovar, then to examine data averaged across replicates of each serovar. Data were submitted to the Gene Expression Omnibus [GEO:GSE11921 and GSE14639].

**Figure 6:**
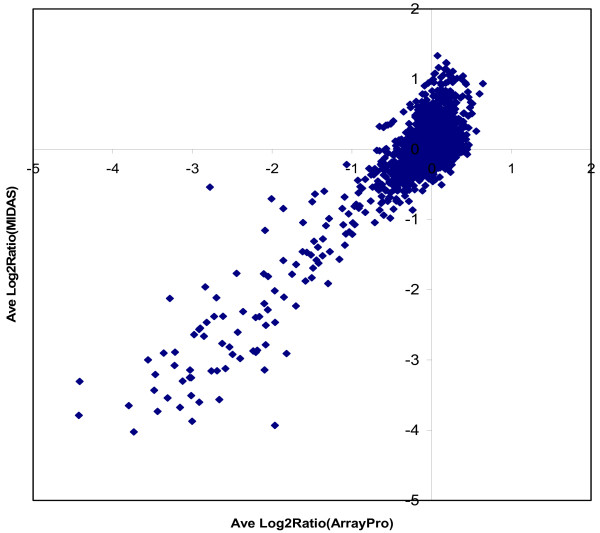
**Comparison between M-CGH Log2Ratio data from *A. pleuropneumoniae *serovar 1 versus serovar 5b hybridizations independently collected, scanned and processed using either ArrayPro software or SpotFinder/MIDAS software**. At least 2 independent hybridizations were performed for each dataset, and the processed normalized data was averaged between replicate experiments and between pairs of duplicate spots on the microarray.

To evaluate M-CGH results obtained using the different methods, we compared M-CGH data from *A. pleuropneumoniae *serovar1 versus serovar 5b hybridizations that were collected, scanned and processed using ArrayPro software or SpotFinder/MIDAS software (Figure 6). Excellent correlation between M-CGH data was obtained using the two different data acquisition and analysis methods.

In cases where more than one reporter on the chip corresponded to a single gene, the data were averaged across the reporters. Visualization and hierarchical clustering of microarray data, using Euclidean Distance metrics and Average Linkage Clustering, was performed in MEV using algorithms developed by Eisen *et al*. [92]. To examine the variation of CGH profiles between the different serovar strains, we generated sample trees as well as support trees based on bootstrapping genes with 1000 iterations. Hierarchical clustering with Manhattan Distance metrics, as well as K-Median Clustering [53] (5 clusters, 1000 iterations) and Cluster Affinity Search Technique [54] (threshold = 0.55) were also performed in MEV to ensure robustness of the clusters.

## Abbreviations

AFLP: Amplified Fragment Length Polymorphism; BHI: Brain Heart Infusion; CPS: Capsular Polysaccharide; LPS: Lipopolysaccharide; M-CGH: Microarray-based Comparative Genomic Hybridization; MLEE: Multilocus Enzyme Electrophoresis; NAD: Nicotinamide Adenosine Dinucleotide; OMP: Outer Membrane Protein; ORF: Open Reading Frame; PFGE: Pulsed-Field Gel Electrophoresis.

## Authors' contributions

JG designed the M-CGH experiments and performed the hybridizations and data analysis with the field isolates. WAF managed the overall bioinformatics, analysed the microarray data from the 15 serovars, and contributed to the design and construction of the microarray. VD contributed to the design of the experiments and participated in the downstream analysis. AB performed the M-CGH hybridizations of the fifteen serovars strains and assisted with the construction of the microarray. SJF assisted with the design of the microarray and the bioinformatics analyses. JIM and JWC participated in the study design and revised the manuscript. JHEN and MJ participated in the conception and supervised the design of the study and revised the manuscript. All authors read and approved the final manuscript.

## Supplementary Material

Additional file 1 **Mean Log2Ratio for genes that are divergent or highly divergent/absent in at least one reference strain of *A. pleuropneumoniae*.** A table listing all the genes that are either divergent or highly divergent/absent in at least one of the 15 reference strains. The number of strains for which a particular gene is identified as divergent or highly divergent/absent is also indicated. Click here for file
