# Supplementary material for: Microarray-based comparative genomic profiling of reference strains and selected Canadian field isolates of Actinobacillus pleuropneumoniae
Source: BMC Genomics. 2009 Feb 24;10:88. doi: 10.1186/1471-2164-10-88 (PMC2653537; doi:10.1186/1471-2164-10-88)
Supplement: Additional file 1 — Mean Log2Ratio for genes that are divergent or highly divergent/absent in at least one reference strain of A. pleuropneumoniae. A table listing all the genes that are either divergent or highly divergent/absent in at least one of the 15 reference strains. The number of strains for which a particular gene is identified as divergent or highly divergent/absent is also indicated. [file 1471-2164-10-88-S1.doc]

**Additional file 1:** Mean Log2Ratio for genes that are divergent or highly divergent/absent in at least one reference strain of *A. pleuropneumoniae*.

|  | **Log2Ratio<-1.0 (divergent)** | | | **Log2Ratio<-3.0 (highly divergent or missing)** | | |
| --- | --- | --- | --- | --- | --- | --- |
| **Gene** | **#Strains** | **Mean Log2Ratio** | **Serovar(s)** | **#Strains** | **Mean Log2Ratio** | **Serovar(s)** |
| APL_0052 | 1 | -1.238 | 3 | 0 | 0 | - |
| APL_0106 | 1 | -1.010 | 8 | 0 | 0 | - |
| APL_0113 | 1 | -1.395 | 3 | 0 | 0 | - |
| APL_0188 | 1 | -1.415 | 14 | 0 | 0 | - |
| APL_0204 | 3 | -1.946 | 3, 4, 15 | 0 | 0 | - |
| APL_0215 | 4 | -1.575 | 8, 9, 10, 11 | 0 | 0 | - |
| APL_0246 | 1 | -1.076 | 14 | 0 | 0 | - |
| APL_0253 | 1 | -1.128 | 15 | 0 | 0 | - |
| APL_0273 | 1 | -1.264 | 8 | 0 | 0 | - |
| APL_0289 | 6 | -1.636 | 2-4, 7, 14, 15 | 0 | 0 | - |
| APL_0290 | 13 | -2.153 | 1-4, 7-15 | 2 | -3.792 | 3, 14 |
| APL_0293 | 1 | -4.161 | 14 | 1 | -4.161 | 14 |
| APL_0313 | 4 | -1.122 | 3, 11, 13, 14 | 0 | 0 | - |
| APL_0328 | 1 | -1.545 | 10 | 0 | 0 | - |
| APL_0329 | 1 | -1.539 | 10 | 0 | 0 | - |
| APL_0330 | 1 | -1.122 | 10 | 0 | 0 | - |
| APL_0341 | 1 | -1.143 | 2 | 0 | 0 | - |
| APL_0355 | 3 | -1.609 | 8, 9, 11 | 0 | 0 | - |
| APL_0356 | 1 | -1.103 | 8 | 0 | 0 | - |
| APL_0357 | 1 | -1.539 | 8 | 0 | 0 | - |
| APL_0363 | 1 | -1.057 | 10 | 0 | 0 | - |
| APL_0387 | 1 | -1.055 | 2 | 0 | 0 | - |
| APL_0442 | 1 | -1.347 | 8 | 0 | 0 | - |
| APL_0488 | 13 | -2.025 | 1-4, 6-12, 14, 15 | 2 | -3.084 | 3, 14 |
| APL_0489 | 13 | -2.662 | 1-4, 6, 8-12, 14-15 | 5 | -3.610 | 2, 3, 12, 14, 15 |
| APL_0490 | 13 | -2.490 | 1-4, 7-15 | 4 | -3.246 | 3, 8, 12, 14 |
| APL_0493 | 14 | -3.279 | 1-4, 5a, 6, 8-15 | 9 | -3.915 | 2, 3, 5a, 8, 9, 11, 12, 14, 15 |
| APL_0494 | 12 | -3.532 | 1-3, 5a, 6, 8-12, 14, 15 | 10 | -3.754 | 2, 3, 5a, 8-12, 14, 15 |
| APL_0495 | 15 | -3.664 | 1-4, 5a, 6-15 | 12 | -4.070 | 2-4, 5a, 6-12, 14, 15 |
| APL_0496 | 15 | -3.648 | 1-4, 5a, 6-15 | 12 | -3.941 | 2-4, 5a, 6-12, 14, 15 |
| APL_0498 | 1 | -1.360 | 5a | 0 | 0 | - |
| APL_0499 | 10 | -2.785 | 2-4, 5a, 6, 7, 12-15 | 2 | -3.759 | 3, 5a |
| APL_0500 | 14 | -2.199 | 2-4, 5a, 6-15 | 2 | -3.458 | 3, 5a |
| APL_0501 | 11 | -2.184 | 2-4, 5a, 6, 7, 10, 12-15 | 1 | -3.320 | 5a |
| APL_0502 | 11 | -3.478 | 2-4, 5a, 6, 7, 10, 12-15 | 8 | -4.043 | 2-4, 5a, 7, 12, 14, 15 |
| APL_0503 | 13 | -2.139 | 2-4, 5a, 6-9, 11-15 | 0 | 0 | - |
| APL_0504 | 15 | -1.921 | 1-4, 5a, 6-15 | 0 | 0 | - |
| APL_0505 | 15 | -3.040 | 1-4, 5a, 6-15 | 9 | -3.567 | 2, 3, 5a, 7-9, 12, 14, 15 |
| APL_0506 | 15 | -2.585 | 1-4, 5a, 6-15 | 4 | -3.539 | 3, 5a, 14, 15 |
| APL_0507 | 14 | -2.318 | 1-4, 5a, 6-9, 11-15 | 1 | -3.708 | 5a |
| APL_0508 | 14 | -3.248 | 1-4, 5a, 6-9, 11-15 | 8 | -3.845 | 2-4, 5a, 7, 12, 14, 15 |
| APL_0509 | 10 | -3.799 | 2-4, 5a, 6, 7, 10, 12, 14, 15 | 9 | -3.950 | 2-4, 5a, 7, 10, 12, 14, 15 |
| APL_0510 | 10 | -4.157 | 2-4, 5a, 6, 7, 10, 12, 14, 15 | 9 | -4.385 | 2-4, 5a, 7, 10, 12, 14, 15 |
| APL_0511 | 10 | -2.627 | 2-4, 5a, 6, 7, 10, 12, 14, 15 | 1 | -4.117 | 5a |
| APL_0512 | 10 | -2.275 | 2-4, 5a, 6, 7, 10, 12, 14, 15 | 1 | -3.974 | 5a |
| APL_0513 | 10 | -2.564 | 2-4, 5a, 6, 7, 10, 12, 14, 15 | 1 | -3.492 | 5a |
| APL_0514 | 10 | -2.454 | 2-4, 5a, 6, 7, 10, 12, 14, 15 | 1 | -3.197 | 5a |
| APL_0515 | 14 | -3.192 | 1-4, 5a, 6-12, 14, 15 | 9 | -3.717 | 2-4, 5a, 7, 10, 12, 14, 15 |
| APL_0516 | 14 | -2.036 | 1-4, 5a, 6-12, 14, 15 | 1 | -3.626 | 5a |
| APL_0517 | 10 | -1.318 | 1-4, 5a, 7, 10, 12, 14, 15 | 0 | 0 | - |
| APL_0518 | 15 | -4.323 | 1-4, 5a, 6-15 | 13 | -4.668 | 1-4, 5a, 7-12, 14, 15 |
| APL_0519 | 4 | -1.158 | 1, 5a, 11, 12 | 0 | 0 | - |
| APL_0520 | 15 | -2.878 | 1-4, 5a, 6-15 | 8 | -3.330 | 3, 4, 8, 10-12, 14, 15 |
| APL_0522 | 14 | -3.871 | 1-4, 5a, 6-12, 14, 15 | 13 | -4.002 | 1-4, 5a, 7-12, 14, 15 |
| APL_0523 | 14 | -3.366 | 1-4, 5a, 6-12, 14, 15 | 13 | -3.456 | 1-4, 5a, 7-12, 14, 15 |
| APL_0524 | 14 | -4.170 | 1-4, 5a, 6-12, 14, 15 | 12 | -4.526 | 1-4, 5a, 8-12, 14, 15 |
| APL_0525 | 14 | -2.231 | 1-4, 5a, 6-12, 14, 15 | 0 | 0 | - |
| APL_0532 | 3 | -1.709 | 3, 10, 14 | 0 | 0 | - |
| APL_0533 | 1 | -1.103 | 3 | 0 | 0 | - |
| APL_0546 | 12 | -1.777 | 1-4, 8-15 | 0 | 0 | - |
| APL_0549 | 1 | -3.769 | 3 | 1 | -3.769 | 3 |
| APL_0550 | 1 | -4.368 | 3 | 1 | -4.368 | 3 |
| APL_0551 | 1 | -4.466 | 3 | 1 | -4.466 | 3 |
| APL_0552 | 1 | -5.131 | 3 | 1 | -5.131 | 3 |
| APL_0553 | 1 | -5.064 | 3 | 1 | -5.064 | 3 |
| APL_0554 | 1 | -2.147 | 3 | 0 | 0 | - |
| APL_0555 | 1 | -4.039 | 3 | 1 | -4.039 | 3 |
| APL_0556 | 2 | -3.760 | 2, 3 | 2 | -3.760 | 2, 3 |
| APL_0557 | 2 | -3.336 | 2, 3 | 2 | -3.336 | 2, 3 |
| APL_0560 | 1 | -2.696 | 3 | 0 | 0 | - |
| APL_0561 | 1 | -1.095 | 3 | 0 | 0 | - |
| APL_0625 | 1 | -1.232 | 10 | 0 | 0 | - |
| APL_0633 | 14 | -2.945 | 1-4, 6-15 | 9 | -3.252 | 1-3, 7-10, 12, 14 |
| APL_0635 | 14 | -3.338 | 1-4, 6-15 | 10 | -3.617 | 2-4, 7-10, 12, 14, 15 |
| APL_0636 | 14 | -3.444 | 1-4, 6-15 | 12 | -3.627 | 1-4, 7-12, 14, 15 |
| APL_0663 | 3 | -1.130 | 8, 10, 11 | 0 | 0 | - |
| APL_0664 | 8 | -2.890 | 1, 2, 8-11, 13, 14 | 2 | -3.396 | 10, 14 |
| APL_0665 | 8 | -2.240 | 1, 2, 8-11, 13, 14 | 0 | 0 | - |
| APL_0666 | 4 | -1.739 | 8, 9, 10, 14 | 0 | 0 | - |
| APL_0670 | 1 | -1.055 | 10 | 0 | 0 | - |
| APL_0676 | 1 | -1.199 | 10 | 0 | 0 | - |
| APL_0697 | 2 | -1.056 | 4, 10 | 0 | 0 | - |
| APL_0703 | 1 | -1.209 | 10 | 0 | 0 | - |
| APL_0705 | 3 | -1.230 | 8, 9, 14 | 0 | 0 | - |
| APL_0784 | 6 | -1.305 | 3, 6, 8-10, 14 | 0 | 0 | - |
| APL_0785 | 9 | -3.259 | 1, 4, 6-9, 11, 13, 14 | 5 | -3.936 | 1, 8, 9, 11, 14 |
| APL_0786 | 8 | -2.477 | 1, 4, 7-9, 11, 13, 14 | 1 | -3.350 | 8 |
| APL_0803 | 1 | -1.018 | 2 | 0 | 0 | - |
| APL_0812 | 2 | -1.842 | 6, 7 | 0 | 0 | - |
| APL_0813 | 1 | -1.755 | 6 | 0 | 0 | - |
| APL_0888 | 1 | -1.082 | 13 | 0 | 0 | - |
| APL_0899 | 5 | -1.059 | 3, 7, 8, 11, 14 | 0 | 0 | - |
| APL_0906 | 3 | -1.265 | 2, 10, 14 | 0 | 0 | - |
| APL_0910 | 4 | -1.213 | 8, 9, 11, 14 | 0 | 0 | - |
| APL_0933 | 2 | -1.999 | 7, 10 | 0 | 0 | - |
| APL_0947 | 2 | -3.202 | 10, 14 | 2 | -3.202 | 10, 14 |
| APL_0948 | 3 | -2.886 | 4, 10, 14 | 2 | -3.309 | 10, 14 |
| APL_0949 | 9 | -2.485 | 1, 4, 7-12, 14 | 2 | -3.819 | 10, 14 |
| APL_0950 | 9 | -1.576 | 1, 4, 7-12, 14 | 0 | 0 | - |
| APL_0952 | 9 | -2.927 | 1, 6-12, 14 | 6 | -3.563 | 1, 8-11, 14 |
| APL_0953 | 7 | -3.095 | 1, 7-11, 14 | 4 | -3.677 | 8-10, 14 |
| APL_0954 | 8 | -2.069 | 1, 7-12, 14 | 2 | -3.389 | 10, 14 |
| APL_0956 | 2 | -4.233 | 10, 14 | 2 | -4.233 | 10, 14 |
| APL_0957 | 2 | -3.552 | 10, 14 | 2 | -3.552 | 10, 14 |
| APL_0979 | 2 | -1.238 | 9, 10 | 0 | 0 | - |
| APL_0980 | 5 | -1.921 | 1, 6, 8, 9, 11 | 0 | 0 | - |
| APL_0981 | 5 | -2.704 | 1, 6, 8, 9, 11 | 2 | -3.481 | 8, 9 |
| APL_0988 | 1 | -1.222 | 8 | 0 | 0 | - |
| APL_0999 | 8 | -1.171 | 2, 3, 7-11, 14 | 0 | 0 | - |
| APL_1038 | 3 | -1.347 | 2, 14, 15 | 0 | 0 | - |
| APL_1069 | 1 | -1.506 | 10 | 0 | 0 | - |
| APL_1093 | 14 | -2.349 | 1-4, 6-15 | 1 | -3.019 | 8 |
| APL_1094 | 14 | -2.795 | 1-4, 6-15 | 6 | -3.274 | 2, 3, 8, 10, 12, 14 |
| APL_1107 | 1 | -1.144 | 15 | 0 | 0 | - |
| APL_1148 | 4 | -1.144 | 3, 7, 8, 14 | 0 | 0 | - |
| APL_1165 | 1 | -1.137 | 10 | 0 | 0 | - |
| APL_1167 | 1 | -1.820 | 10 | 0 | 0 | - |
| APL_1182 | 14 | -2.825 | 1-4, 6-15 | 6 | -3.485 | 1, 8-11, 14 |
| APL_1183 | 14 | -2.233 | 1-4, 6-15 | 1 | -3.075 | 9 |
| APL_1184 | 14 | -2.377 | 1-4, 6-15 | 1 | -3.222 | 10 |
| APL_1185 | 12 | -1.656 | 1-4, 7-12, 14, 15 | 0 | 0 | - |
| APL_1193 | 14 | -3.496 | 1-4, 6-15 | 12 | -3.743 | 1-4, 7-12, 14, 15 |
| APL_1194 | 14 | -3.833 | 1-4, 6-15 | 12 | -4.038 | 1-4, 7-12, 14, 15 |
| APL_1195 | 14 | -4.980 | 1-4, 6-15 | 13 | -5.145 | 1-4, 7-15 |
| APL_1196 | 14 | -4.088 | 1-4, 6-15 | 13 | -4.192 | 1-4, 7-15 |
| APL_1197 | 4 | -1.069 | 7, 8, 10, 11 | 0 | 0 | - |
| APL_1199 | 2 | -1.017 | 7, 12 | 0 | 0 | - |
| APL_1200 | 11 | -2.253 | 1-4, 7-9, 11-13, 15 | 0 | 0 | - |
| APL_1201 | 12 | -3.481 | 1-4, 6-9, 11-13, 15 | 9 | -3.820 | 1-4, 7-9, 11, 12 |
| APL_1202 | 12 | -3.160 | 1-4, 6-9, 11-13, 15 | 8 | -3.537 | 1-4, 8, 9, 11, 12 |
| APL_1218 | 1 | -1.335 | 2 | 0 | 0 | - |
| APL_1227 | 1 | -1.186 | 9 | 0 | 0 | - |
| APL_1231 | 1 | -1.084 | 8 | 0 | 0 | - |
| APL_1235 | 2 | -1.489 | 3, 10 | 0 | 0 | - |
| APL_1256 | 1 | -1.601 | 15 | 0 | 0 | - |
| APL_1262 | 1 | -1.062 | 9 | 0 | 0 | - |
| APL_1299 | 2 | -3.360 | 10, 14 | 2 | -3.360 | 10, 14 |
| APL_1301 | 10 | -1.835 | 1-4, 7-9, 11, 13, 15 | 0 | 0 | - |
| APL_1304 | 5 | -1.251 | 3, 7, 8, 11, 14 | 0 | 0 | - |
| APL_1329 | 5 | -2.293 | 2-4, 6, 15 | 1 | -3.081 | 3 |
| APL_1330 | 3 | -1.226 | 2, 3, 4 | 0 | 0 | - |
| APL_1350 | 1 | -1.562 | 8 | 0 | 0 | - |
| APL_1351 | 2 | -1.115 | 8, 11 | 0 | 0 | - |
| APL_1352 | 1 | -1.035 | 8 | 0 | 0 | - |
| APL_1376 | 1 | -1.066 | 7 | 0 | 0 | - |
| APL_1402 | 2 | -1.140 | 8, 11 | 0 | 0 | - |
| APL_1442 | 1 | -2.276 | 3 | 0 | 0 | - |
| APL_1443 | 1 | -4.106 | 3 | 1 | -4.106 | 3 |
| APL_1444 | 8 | -3.181 | 2, 3, 4, 6, 7, 12, 13, 15 | 4 | -3.805 | 2, 3, 7, 12 |
| APL_1445 | 8 | -2.083 | 2, 3, 4, 6, 7, 12, 13, 15 | 1 | -3.261 | 3 |
| APL_1460 | 12 | -2.088 | 1, 3, 4, 7--15 | 1 | -3.027 | 8 |
| APL_1461 | 2 | -1.305 | 3, 14 | 0 | 0 | - |
| APL_1463 | 13 | -2.102 | 1-4, 7-15 | 0 | 0 | - |
| APL_1464 | 14 | -2.810 | 1-4, 6-15 | 3 | -3.503 | 3, 8, 11 |
| APL_1465 | 14 | -2.881 | 1-4, 6-15 | 8 | -3.311 | 1-4, 8, 10, 12, 14 |
| APL_1466 | 13 | -2.326 | 1-4, 7-15 | 2 | -3.500 | 8, 9 |
| APL_1467 | 14 | -3.189 | 1-4, 6-15 | 9 | -4.011 | 1-4, 7-9, 11, 15 |
| APL_1468 | 12 | -3.202 | 1-4, 6-9, 11-13, 15 | 7 | -3.622 | 1, 3, 4, 7, 8, 11, 15 |
| APL_1469 | 14 | -2.721 | 1-4, 6-15 | 8 | -3.297 | 1-4, 7-9, 11 |
| APL_1470 | 14 | -2.750 | 1-4, 6-15 | 6 | -3.652 | 1-3, 8, 9, 15 |
| APL_1471 | 11 | -3.734 | 1-4, 6-9, 12, 13, 15 | 10 | -3.928 | 1-4, 7-9, 12, 13, 15 |
| APL_1472 | 12 | -2.388 | 1-4, 6-9, 11-13, 15 | 1 | -3.420 | 8 |
| APL_1506 | 1 | -1.129 | 8 | 0 | 0 | - |
| APL_1568 | 13 | -2.020 | 1-4, 7-15 | 1 | -3.240 | 8 |
| APL_1573 | 1 | -2.013 | 2 | 0 | 0 | - |
| APL_1575 | 5 | -1.992 | 1, 4, 12, 14, 15 | 0 | 0 | - |
| APL_1576 | 13 | -2.498 | 1-4, 6-9, 11-15 | 2 | -3.075 | 3, 14 |
| APL_1577 | 13 | -2.695 | 1-4, 6-9, 11-15 | 4 | -3.118 | 1, 3, 8, 14 |
| APL_1578 | 4 | -1.104 | 2, 3, 8, 11 | 0 | 0 | - |
| APL_1579 | 14 | -3.449 | 1-4, 6-15 | 12 | -3.691 | 1-4, 7-12, 14, 15 |
| APL_1580 | 14 | -3.076 | 1-4, 6-15 | 7 | -3.736 | 2, 3, 8-10, 12, 14 |
| APL_1581 | 14 | -2.792 | 1-4, 6-15 | 6 | -3.152 | 2, 3, 8-10, 12 |
| APL_1582 | 7 | -1.177 | 3-4, 7-10, 12 | 0 | 0 | - |
| APL_1583 | 4 | -1.836 | 2-4, 15 | 0 | 0 | - |
| APL_1584 | 3 | -2.115 | 4, 10, 15 | 0 | 0 | - |
| APL_1585 | 2 | -1.327 | 4, 15 | 0 | 0 | - |
| APL_1621 | 1 | -1.295 | 15 | 0 | 0 | - |
| APL_1622 | 1 | -1.015 | 15 | 0 | 0 | - |
| APL_1677 | 3 | -1.147 | 3, 4, 8 | 0 | 0 | - |
| APL_1678 | 5 | -1.187 | 2, 7-9, 11 | 0 | 0 | - |
| APL_1680 | 11 | -3.123 | 1-4, 6-9, 11, 12, 15 | 7 | -3.510 | 1-3, 7-9, 11 |
| APL_1681 | 13 | -3.309 | 1-4, 7-15 | 10 | -3.526 | 1-3, 7-12, 14 |
| APL_1683 | 1 | -1.299 | 10 | 0 | 0 | - |
| APL_1690 | 2 | -1.234 | 8, 11 | 0 | 0 | - |
| APL_1691 | 1 | -1.027 | 3 | 0 | 0 | - |
| APL_1692 | 4 | -1.173 | 1, 3, 10, 14 | 0 | 0 | - |
| APL_1706 | 1 | -1.290 | 10 | 0 | 0 | - |
| APL_1707 | 3 | -2.257 | 3, 4, 10 | 1 | -3.181 | 10 |
| APL_1708 | 6 | -1.166 | 1-3, 8, 9, 11 | 0 | 0 | - |
| APL_1729 | 11 | -3.061 | 1-4, 8, 9, 11-15 | 7 | -3.333 | 1, 3, 8, 9, 11, 12, 14 |
| APL_1733 | 4 | -1.573 | 1, 8, 9, 11 | 0 | 0 | - |
| APL_1734 | 8 | -1.945 | 1, 3, 4, 8-12 | 0 | 0 | - |
| APL_1745 | 4 | -1.337 | 2, 8, 11, 12 | 0 | 0 | - |
| APL_1746 | 5 | -1.204 | 2, 8, 11, 12, 14 | 0 | 0 | - |
| APL_1748 | 8 | -1.826 | 1-3 8, 9, 11, 12, 15 | 0 | 0 | - |
| APL_1869 | 4 | -1.714 | 2, 7, 9, 14 | 0 | 0 | - |
| APL_1904 | 1 | -1.173 | 9 | 0 | 0 | - |
| APL_1963 | 5 | -1.194 | 3, 7, 8, 11, 14 | 0 | 0 | - |
| APL_1980 | 13 | -2.035 | 1-4, 7-15 | 0 | 0 | - |
| APL_2016 | 14 | -2.334 | 1-4, 6-15 | 0 | 0 | - |
| APL_2023 | 10 | -1.634 | 1-3, 7-11, 14, 15 | 0 | 0 | - |
| APL_2024 | 9 | -3.057 | 1-3, 7-11, 14, 15 | 6 | -3.279 | 3, 7, 8, 10, 11, 14 |
| APL_2025 | 1 | -1.003 | 7 | 0 | 0 | - |
| APL_2045 | 10 | -3.015 | 1, 2, 7-9, 11-15 | 7 | -3.266 | 2, 7-9, 11, 12, 14 |
| APL_2046 | 10 | -2.846 | 1, 2, 7-9, 11-15 | 5 | -3.194 | 2, 8, 9, 11, 14 |
